# Corneal Optical Quality Following Sub 1.8 mm Micro-Incision Cataract Surgery vs. 2.2 mm Mini-Incision Coaxial Phacoemulsification

**DOI:** 10.4103/0974-9233.61225

**Published:** 2010

**Authors:** Jorge L. Alió, Bassam Elkady, Dolores Ortiz

**Affiliations:** VISSUM, Instituto Oftalmologico de Alicante, Lecturer of Ophthalmology, Ain Shams University, Cairo, Egypt; 1 VISSUM, Instituto Oftalmologico de Alicante, Spain; 2 Miguel Hernandez University, Alicante, Spain

**Keywords:** Micro-Incision Cataract Surgery, Micro-Coaxial Phacoemulsification

## Abstract

**Purpose:**

To study and compare the effects of the micro-incision cataract surgery (MICS-sub 1.8 mm) and miniincision coaxial phacoemulsification (2.2 mm) on the optical quality of the cornea characterized in terms of corneal aberrations.

**Materials and Methods:**

Fifty eyes underwent MICS and 50 mini-incision phacoemulsification, by the same surgeon. Both types of cataract surgery were performed using low ultrasound power and through a clear corneal incision, placed on the steepest corneal meridian ranging from 1.6 to 1.8 in MICS (Group I) and from 2.12 to 2.3 mm in mini-incision coaxial phacoemulsification (Group II). Seidel and Zernike aberration coefficients and RMS values were obtained for a 6-mm pupil preoperatively and one month after surgery.

**Results:**

The corneal astigmatism did not show statistically significant changes in either of the two groups: (MICS: –0.73 ± 0.63, –0.65 ± 0.53 D, *P* = 0.25), (mini-incision phacoemulsification; –1.21 ± 1.52, –1.00 ± 1.19 D, *P* = 0.12). The total RMS remained unchanged after MICS (1.77 ± 1.7, 1.65 ± 1.3 μm, *P* = 0.18) and mini-incision phacoemulsification (2.00 ± 1.87, 2.09 ± 1.8 μm, *P* = 0.41). Statistically significant changes were found for coma (P = 0.004) and higher-order aberrations (*P* < 0.001), showing MICS significantly less changes in cornea.

**Conclusions:**

Both MICS and mini-incision phacoemulsification do not degrade the optical quality of the cornea. Both surgeries do not induce a modification of the corneal astigmatism, even in the axis. It seems that 2 mm is the limit around which no optical changes are induced by cataract surgery in the human cornea.

## INTRODUCTION

There is a definite trend nowadays towards the reduction and even elimination of the corneal consequences of cataract surgery through reducing the incision size not only to decrease the incidence of wound leak and postoperative infection,^1^,^2^ but also aiming to gain all the corneal optical advantages of small incision size cataract surgery to better preserve corneal optical performance. This is especially important when considering modern special lenses such as Toric, aberration correcting and others.^1^,^3^–^6^

Corneal refractive changes following cataract surgery are related to the location and size of the corneal incision. Therefore, smaller incision can manipulate the cornea more gently with minimal stress and injury to the surrounding tissues providing better optical outcomes,^6^–^8^ through having a corneal incision of a good quality, both optically and morphologically.^5^,^9^ A significantly better control of astigmatism has been demonstrated related to the reduction of corneal incision size for over 3 mm to less than 2 mm,^6^–^8^ resulting in a reduction of surgically induced astigmatism (SIA) and corneal aberrations.^6^–^8^

Nowadays, the smallest incision available is that of micro-incision co-axial cataract surgery (MICS), defined as sub 1.8 mm incisional cataract surgery; larger bi-axial incisions than 2 mm are called mini-incision phacoemulsification. Previous studies have demonstrated the better control of astigmatic and aberrometric corneal changes that happen in MICS.

However, there is no published reference on the relative advantages of sub 1.8 mm incisions (MICS) over mini-incisions of about 2.2 mm. As the demonstration of differences may lead to important decisions by surgeons, concerning the transition towards incisions smaller than 2 mm, a further clarification of whether such differences exist, seems to be mandatory.^5^,^11^–^16^

The aim of this study is to investigate the effects of the sub 1.8 mm co-axial micro-incision cataract surgery (MICS) and mini-incisions (2.2 mm) co-axial phacoemulsification on the optical quality of the cornea characterized in terms of the corneal aberrations.

## MATERIALS AND METHODS

### Study design

Comparative, consecutive, prospective, interventional, clinical trial.

### Patients

A hundred eyes of 100 patients with cataracts grade II–IV (Lens Opacities Classification System [LOCS]III),^17^ were enrolled in this study. Patients were interviewed for demographic data, confirmation of ocular, systemic and medical histories. There were 53 females and 47 males with a mean age of 67.86 years (ranging from 53 to 89 years).

Inclusion criteria: Age range between 40 and 90 years, no history of eye surgery or glaucoma, absence of corneal disease of any type, pupil dilation at the preoperative examination of at least 7 mm.

Exclusion criteria: Cataracts graded as more than grade 4 (Lens Opacities Classification System III),^17^ eyes with >3 diopters (D) of regular astigmatism or significant irregular astigmatism. All patients signed an adequate informed consent. The study followed the tenets of the Declaration of Helsinki stated in 2004 (World Medical Association Declaration of Helsinki, Ethical Principles for Medical Research Involving Human Subjects, 52^nd^ General Assembly, Edinburgh, Scotland, October 2000; amended 2004. Available at: http://www.wma.net/e/policy/b3.htm Accessed September 23, 2004). The Ethics committee of our institution approved the study.

Uneventful phacoemulsification surgery and intraocular lens (IOL) implantation were performed on all patients using either micro-incision bi-axial sub 1.8 mm lens surgery by the same surgeon (JLA). The patients were randomly assigned into two groups, depending upon the type of the surgery performed [Table 1]:

**Table 1:** Patient's demographic data

	MICS	Microphaco
Intended incision size (mm)	1.6	2.2
Capsulorrhexis diameter (mm)	5.5	5.5
Phacoemulsification tip	30° (micro tip)	30° (micro tip)
Phacoemulsification tip calibre	0.9	0.9
Prechopping	Yes	Yes
Power	20-30%	20-30%
Aspiration flow	25 cm^3^/min	25 cm^3^/min
Vacuum	550 mmHg	550 mmHg
Anterior chamber pressure	90 cm^3^-H_2_O	90 cm^3^-H_2_O
Mode	Hyperpulser,	Hyperpulser,
	80 ms, 40%	80 ms, 40%
Irrigation/aspiration of the cortical remnants	450 mmHg	450 mmHg
Irrigation/aspiration of viscoelastic	40 mmHg	40 mmHg

Group I (50 eyes of 50 patients) underwent sub 1.8 mm bi-axial micro-incision phacoemulsification (MICS) targeting an incision of sub-1.8 mm.

Group II (50 eyes of 50 patients) underwent co-axial phacoemulsification through mini-incision of 2.2 mm.

Preoperative assessment consisted of a full standard comprehensive ophthalmic examination including: Clinical data, refractive status of the eye, uncorrected and best spectacle corrected distance visual acuities (UCDVA and BSCDVA), slit-lamp examination, intraocular pressure measurement (IOP) by Goldmann applanation tonometry, cataract grading (LOCS III System), indirect binocular ophthalmoscopy of the eye fundus and corneal topography and corneal aberrometry (CSO topographer, Firence, Italy).

All postoperative follow-ups were carried out by an independent observer (BAK), different from the surgeon. Postoperative visits included: Slit-lamp biomicroscopic evaluation, applanation tonometry and UCDVA and BSCDVA. Corneal topography and aberrometry was performed 1 month, postoperatively.

### Surgical technique

Surgery was performed in all cases by the same surgeon (JLA), using sutureless phacoemulsification techniques. Topical anesthesia with 2% preservative-free lidocaine (B. Braun, Barcelona, Spain) was used in all cases, with mild sedation with midazolam (Roche, Madrid, Spain). Adequate dilatation was obtained with intra-cameral mydriasis using 1 mL of a vial containing cyclopentolate 1% 1 ml, Phenylephrine 10% 1.5 mL, Lignocaine 2% 5 mL and balance saline solution (BSS).

### Biaxial sub 1.8 mm micro-incision cataract surgery

Micro-incision cataract surgery (MICS) was performed in 50 eyes (Group I), a 1.4 mm clear corneal incision was made at the steepest corneal meridian using an Alio MICS diamond blade (Katena, Denville, NJ). This incision was the one used also for the IOL implantation. A second similar clear corneal incision was made at 90°. An Infiniti phacoemulsification platform (Alcon Laboratories, Forth Worth, TX, USA) was used in MICS. Surgery was performed using a phacoemulsification tip of 30° (micro tip), 0.9 caliper with pre-chopping and hyper-pulse (80 ms, 40%) mode. MICS surgery was performed following a previously described protocol.^1^,^2^,^6^

### A 2.2 mm Co-axial mini-incisions phacoemulsification (Mini- incision phacoemulsification)

Mini-incision phacoemulsification was performed in 50 eyes (Group II), a 2.2 mm clear corneal micro-incision was placed on the steepest corneal meridian using a stainless steel microkeratome. A paracentesis incision of 1 mm was made 90° apart with a calibrated knife (Alcon). After the capsulorrhexis was done, hydrodissection, nucleus rotation, prechopping and phacoemulsification was done using the Infiniti Vision System involving a technique similar to that used on group I. The only difference is that we used the mechanism of ‘gas-forced infusion’ as a pump to pressurize inflow of fluidics for the MICS group for optimum anterior chamber stability and not for the mini-incision phacoemulsification group. Table 2 illustrates surgical settings for both groups.

**Table 2:** Surgical parameters used in the study with the infiniti phacoemulsification platform. ‘Gas forced infusion’ pump was used in micro-incision co-axial cataract surgery

	MICS	Microphaco
No. of patients	36	22
No. of eyes	44	33
Female patients No/%	19 (52.78)	13 (59.09)
Male patients No/%	17 (47.22)	9 (40.91)
Mean age (y) ± SD	70.75 ± 8.97	68.14 ± 7.36
Range of age (y)	54-90	53-80

### Intraocular lens implantation

An Acri.Smart 48S lens (Acri.Tec GmbH, Hennigsdorf, Germany) was implanted in all cases. Acri.Smart 48S is a monofocal foldable MICS IOL. In the MICS group, the incision was enlarged laterally at its internal side to a maximum of 1.8 mm with a calibrated diamond blade (Katena). The tip of the cartridge was introduced partially into the external part of the incision, and that was followed by the injection of the lens in the capsular bag. The proximal part was placed into the bag with a second instrument, which was inserted through the second incision (Alio intraocular manipulator, Katena). After the removal of the ophthalmic viscoelastic device, the incisions were hydrated using a 30-gauge cannula (Alcon), in both groups. Intraocular preservative-free antibiotic (0.1 cm^3^; cefuroxime 1%, Glaxo SmithKline, Madrid, Spain) was injected into the anterior chamber in all cases. No stitches were used in any of the cases in either group. Postoperative topical therapy included a combination of topical antibiotics [EXOCIN^®^ eye drops (Ofloxacin 0.3%)] and steroid [MAXIDEX^®^ eye drops (Dexamethasone alcohol 0.1%) Alcon Cusi, Barcelona-Spain].

The corneal incision length was measured at the end of the procedure using the gauge caliper (Memmen-Steinert, ASICO), which measures from 1.00 to 4.00 mm in 0.10 mm increments. The tips of the gauge caliper were applied to the inner lips of the incision to measure a moment in which the caliper could not enter the anterior chamber.

### Corneal aberrometry

Corneal aberrations were derived from the data of the anterior surface of the cornea obtained from a topography system: The CSO topographer (CSO, Firenze, Italy). The software of this topography system, the EyeTop2005 (CSO, Firenze, Italy), makes the conversion of the corneal elevation profile into corneal wavefront data using the Zernike polynomials with an expansion up to the seventh order. This topography system analyses a total of 6144 corneal points from a corneal zone between 0.33 and 10 mm with respect to the corneal vertex.

To investigate changes in corneal aberrations, the root mean square (RMS) of the wave aberration (Seidel aberrations) for total, coma (Z3 ± 1), spherical (Z40), reported with its sign, astigmatism (Z2 ± 2) and higher aberrations (HOA) at 6-mm pupil diameter were studied 1 month, postoperatively.

### Statistical analysis

SPSS statistics software package version 10.1 for Windows (SPSS, Chicago, Illinois, USA) was used for statistical analysis. Paired *t*-test was used for comparison between pretreatment and 1 month data. Statistical tests were performed at the 95% confidence interval.

### Main outcome measures

Changes in corneal astigmatism measured by corneal topography and corneal aberrations changes as measured by corneal aberrometry described in terms of Zernike polynomials and Seidel aberrations.

## RESULTS

In Group I, all incisions ranged from 1.6 to1.8 mm. In Group II, all incisions ranged from 2.15 to 2.3 mm and no incision was longer than 2.3 mm, with a statistically significant difference between groups (1.69 ± 0.12 versus 2.25 ± 0.06; *P* < 0.001).

**Figure 1:**
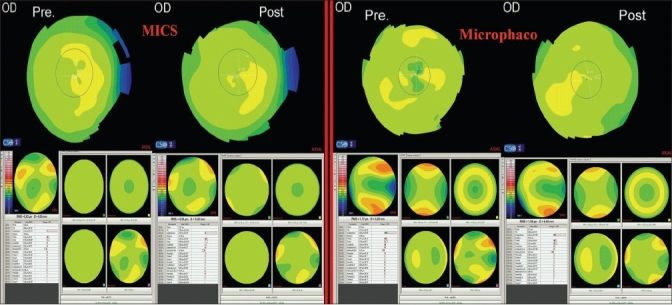
Top: Corneal topography maps (axial maps) preoperative versus 1 month after surgery for one eye from each group of the study. Bottom: Corneal aberrometry maps (Seidel panel) preoperative versus 1 month after surgery of the same eyes

**Figure 2:**
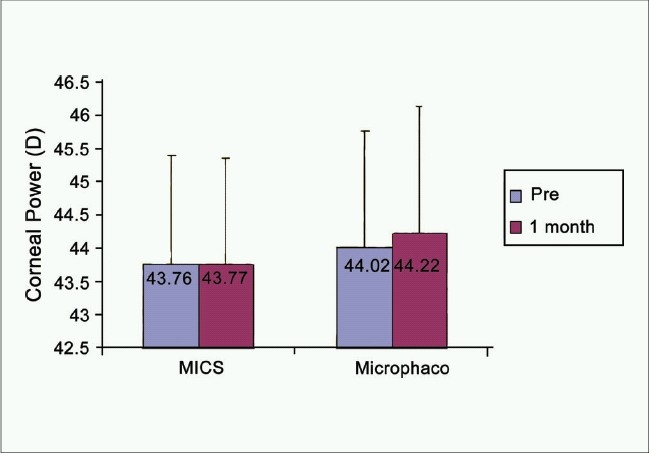
Corneal power in diopters, pre versus. 1 month postoperative, in both groups

**Figure 3:**
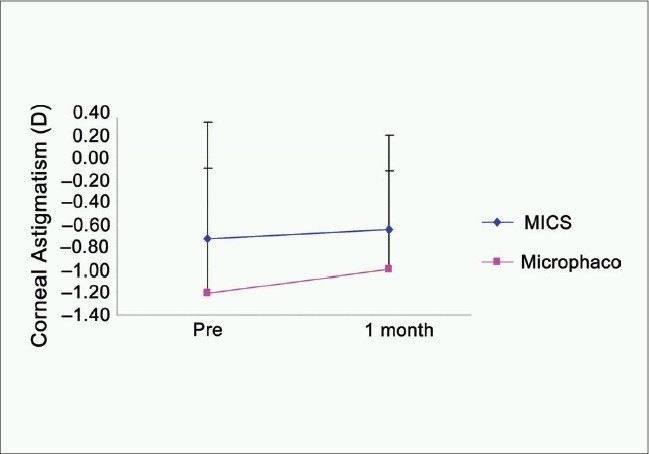
Corneal astigmatism in dioptres, pre versus. 1 month postoperative, in both groups

The comparison between corneal topography and aberrometry maps preoperatively versus 1 month postoperatively for both groups is shown in Figure 1. The corneal power slightly changed after surgery in both groups [Figure 2]; (MICS: 43.76 ± 1.63 D versus 43.77 ± 1.58 D), (mini-incision phacoemulsification; 44.02 ± 1.74 D versus 44.22 ± 1.92 D). No statistically significant differences were found pre- and postoperatively in either of the groups, (*P* = 0.93, *P* = 0.1), respectively. In both groups, the corneal astigmatism [Figure 3] showed some changes, but did not show levels of statistical significance; (MICS: –0.73 ± 0.63 D versus –0.65 ± 0.53 D, *P* = 0.25), (Mini-incision phacoemulsification; –1.21 ± 1.52 D versus –1.00 ± 1.19 D, *P* = 0.12).

**Figure 4:**
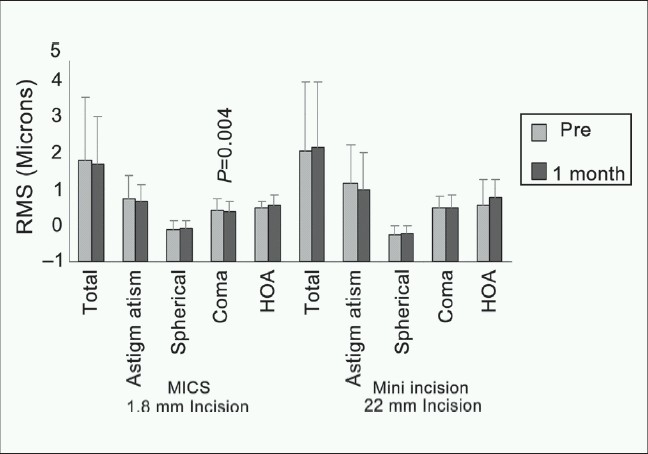
Corneal aberrations (Seidel coefficients) at 6-mm pupil diameter before and 1 month after surgery, in both groups

The evolution of the corneal aberrations after surgery is shown in Figure 4. The RMS value of the total corneal aberrations slightly decreased on average after MICS (pre, 1.77 ± 1.7 µm; 1 month post, 1.65 ± 1.3 µm) and slightly increased after mini-incision phacoemulsification (pre, 2.00 ± 1.87 µm; 1 month post, 2.09 ± 1.8 µm). However, the difference was not statistically significant in either of the groups, MICS (*P* = 0.18), mini-incision phacoemulsification (*P* = 0.41).

Analysis of individual Zernike terms for astigmatism, spherical, coma and higher order aberrations (HOA) showed as follows: Group 1: Mean astigmatism 0.7 ± 0.65 µm preoperatively and 0.65 ± 0.45 µm at 1 month postoperatively, *P* = 0.52, Group II: 1.14 ± 1.05 µm preoperatively and 0.96 ± 1.01 µm at 1 month postoperatively, *P* = 0.11, spherical and spherical-like aberrations (Seidel) Group I pre, –0.12 ± 0.23 µm; 1 month post, –0.1 ± 0.21 µm, *P =* 0.4, Group II, pre, –0.25 ± 0.22 µm; 1 month post, –0.23 ± 0.19 µm, *P* = 0.19. Coma and coma-like aberration; decreased in Group I (MICS); pre, 0.41 ± 0.29 µm; 1 month post, 0.35 ± 0.28 µm with a statistically significant difference, *P* = 0.004. In Group II (mini-incision phacoemulsification); no statistically significant difference were found: Pre, 0.46 ± 0.31 µm; 1 month post, 0.47 ± 0.33 µm, *P* = 0.61. The mean HOA was: In Group I: Pre, 0.45 ± 0.19 µm; 1 month post, 0.53 ± 0.29 µm, *P* = 0.01, while in Group II, pre, 0.54 ± 0.68 µm; 1 month post, 0.75 ± 0.48 µm, *P* = 0.01, with no statistically significant difference in either groups.

## DISCUSSION

Optical outcomes after cataract surgery depend on two main factors combined together to produce the fine retinal image, which are the eye's aberrations (modified by the surgery) and IOL-induced aberrations.^18^–^20^ Incision size has the greatest impact on the optical aberrations induced by surgery, and the smaller the incision, the lower the aberrations, the better the optical quality.^1^,^8^,^20^,^21^

We have reported the evidence that MICS incision is superior to standard co-axial phacoemulsification as regard corneal aberration induction.^6^,^10^ Furthermore, we have also reported^5^ that the reduction in incisions sizes does not depend on the quality of the corneal incision, studied by corneal optical coherence tomography.

In this report, we used corneal aberrometry to evaluate corneal optical quality, since about 80% of all aberrations of the human eye occur in the corneal surface.^22^ Corneal aberrometry is also more sensitive and reliable than global aberrometry for the purposes of this study. The topography system we used (CSO topographer) analyses up to 6144 corneal points within corneal zone between 0.33 and 10 mm with respect to corneal vertex, with more expressive analysis and assessment for up to the seventh order aberration by using the Zernike polynomials. In addition, it avoids superimposing of the light spots associated to the different parts of the wavefront produced by a highly aberrated eye. Finally, with some kinds of global aberrometry it is assumed that the slope of the wavefront in each analysed portion is locally flat; this could induce significant errors in the calculated final results.^22^

Changes in corneal aberrations after different techniques of cataract surgery have been the subject discussed previously by many authors;^7^,^10^,^23^–^25^ in the present study, we have targeted the demonstration of the effect that two types of incisions show on the corneal optical performance after surgery with the aim of ascertaining which are the real limits in cataract incision size to obtain an aberrometric and astigmatic corneal refractive outcome after cataract surgery.

Our results showed that although the cornea showed some astigmatic changes, they were not statistically significant when 1.8 and 2.2 mm incisions were compared. So we conclude that both micro (1.8 mm) and mini-incision (2.2mm) phacoemulsification were able to provide an astigmatically neutral incision.^6^

Analysis of the individual Zernike terms showed that there were no statistically significant changes in any of the individual aberrations evaluated (astigmatism, spherical, coma and HOA) postoperatively in both study groups, with the exception of only minor postoperative reduction in the limits of statistical significance as coma aberration in favor of MICS. This finding supports our previous observation with MICS^6^ and is in agreement with other studies.^7^,^8^ All the values of individual aberration, except HOA, slightly changed on average postoperatively with no statistically significant differences in both groups.

Therefore, we can conclude that surgery performed through either incision size (1.8 and 2.2 mm), behave similarly concerning postoperative corneal astigmatism and HOA and, therefore offer similar advantages.

## CONCLUSION

Both MICS sub 1.8 mm incision and mini-incision coaxial phacoemulsification 2.2 mm incision are equally effective in preserving the corneal optics after surgery. It seems that around 2 mm incisions are the limit for the induction of nonsignificant optical changes in the human cornea following cataract surgery.
